# Progressive ontogenetic niche shift over the prolonged immaturity period of wandering albatrosses

**DOI:** 10.1098/rsos.171039

**Published:** 2017-10-11

**Authors:** Alice Carravieri, Henri Weimerskirch, Paco Bustamante, Yves Cherel

**Affiliations:** 1 Centre d'Etudes Biologiques de Chizé, UMR 7372 CNRS-Université de La Rochelle, 79360 Villiers-en-Bois, France; 2 Littoral Environnement et Sociétés (LIENSs), UMR 7266 CNRS-Université de la Rochelle, 2 rue Olympe de Gouges, 17000 La Rochelle, France

**Keywords:** stable isotopes, mercury, moult, feeding ecology, subtropics, subantarctic

## Abstract

Very little is known about trophic ontogenetic changes over the prolonged immaturity period of long-lived, wide-ranging seabirds. By using blood and feather trophic tracers (δ^13^C and δ^15^N, and mercury, Hg), we studied age-related changes in feeding ecology during the immature phase of wandering albatrosses *Diomedea exulans* when they gradually change from a pure oceanic life to visits to their future breeding grounds. Immatures fed in subtropical waters at high trophic positions during moult. Between- and within-individual variations in isotopic niche were very high, irrespective of age, highlighting wide-ranging exploratory behaviours. In summer, while acting as central-place foragers from their future breeding colony, individuals progressively relied on lower trophic level prey and/or southern latitudes as they aged, until occupying a similar isotopic niche to that of adults. Immatures had exceptionally high Hg burdens, with males having lower Hg concentrations than females, suggesting that they foraged more in subantarctic waters. Our findings suggest a progressive ontogenetic niche shift during central-place foraging of this long-lived species.

## 1. Introduction

In long-lived species, young individuals have a critical impact on population dynamics through their survival and recruitment rates [1]. The immaturity stage may last several years, with the first breeding attempt occurring well after physiological maturity. Young individuals may need a long learning period to increase their foraging skills and body condition [2] before being recruited into the breeding population [3]. This might be exacerbated in marine environments, where prey occurrence is patchy and difficult to predict, thus involving complex foraging skills. As such, a failure in optimizing food acquisition leads to mortality in seabirds during the first months of independence [1]. Ontogenetic changes in feeding ecology over the immaturity stage have been rarely quantified [4]. Yet, they could be critical in shaping how immatures become adults, and explaining their prolonged breeding deferral.

Feeding ecology of immature seabirds is largely unknown, with diet information being biased towards chicks and breeding adults [4]. Lack of information is especially significant in species displaying large-scale movement strategies and prolonged immaturity such as albatrosses, which spend this period almost exclusively at sea. For instance, juvenile wandering albatrosses *Diomedea exulans* have a pure oceanic life for 2 years at least (average 5 years) after leaving their birth place [3]. Then, in order to search for future mates, they visit their natal colonies as immatures for short periods. During these visits, immatures are central-place foragers, and have either to compete with adults or to exploit different habitats [5] and/or prey. Over years, immatures could learn to use the waters surrounding the colony and gradually change their feeding behaviour to match that of adults (progressive ontogenetic shift hypothesis [4]).

Here, we quantify age-related differences in feeding ecology in immature wandering albatrosses from the Crozet Islands (southern Indian Ocean) by using trophic tracers (δ^13^C and δ^15^N, and mercury, Hg). Trophic information was obtained over two temporal scales: when immatures disperse widely and moult over oceanic waters (feathers), and when they behave as central-place foragers during visits to the colony (blood). Under the progressive ontogenetic shift hypothesis [4], we expected trophic tracer values to change gradually with age until becoming similar to those of adults.

## 2. Material and methods

Thirty-nine immature wandering albatrosses were sampled in January–March 2015 on Possession Island, Crozet Archipelago (46°S, 52°E), where a long-term capture–mark–recapture programme of the entire population started in 1966 [3]. Individuals were 3–8 years old and had never attempted reproduction. Blood (1.5 ml) was taken from the tarsal vein with 2 ml syringes and kept in ethanol (70%) until analyses. One dorsal body feather was sampled from the lower back and 1–2 cm sections of barbs were cut from primary feathers. Wandering albatrosses have a protracted wing moult pattern, with up to three generations of primaries being present simultaneously and identifiable by their degree of wear: new (not abraded, less than or equal to 1 year old), intermediate (abraded, 1–2 years old), and old (very abraded, more than or equal to 2 years old) feathers [6,7]. The bird age at feather synthesis was therefore deduced. For example, in a 5-year-old individual a new primary (and body feather) was assigned age 4, an intermediate primary age 3 and an old primary age 2 [6,7]. Feather moult occurs entirely outside the breeding period [6,7]. In seven individuals (aged 3–5), primaries of large chicks close to fledging were still present, as shown by their low δ^13^C, δ^15^N and Hg values (‘chick feathers’, table 1). These feathers were excluded from further analyses.

**Table 1.** Feather and blood δ^13^C, δ^15^N and Hg values in immature wandering albatrosses from Crozet Islands.

	*N*	δ^13^C (‰) mean ± s.d. [min; max]	δ^15^N (‰) mean ± s.d. [min; max]	Hg (µg g^−1^ dw) mean ± s.d. [min; max]
	feathers^a^			
F	22	−16.6 ± 0.4 [−17.8; −16.0]	17.3 ± 0.5 [16.2; 18.2]	42.9 ± 9.5 [28.1; 65.1]
M	17	−16.7 ± 0.4 [−17.8; −15.8]	17.2 ± 0.4 [16.4; 17.9]	33.1 ± 6.7 [25.0; 51.4]
chicks	7	−20.0 ± 0.5 [−20.5; −19.3]	14.9 ± 0.4 [14.3; 15.5]	8.6 ± 1.5 [6.2; 10.4]
	blood			
F	22	−20.0 ± 0.6 [−21.1; −18.8]	14.4 ± 0.4 [13.2; 15.3]	11.0 ± 3.6 [5.0; 19.4]
M	17	−20.3 ± 0.5 [−21.6; −19.2]	14.0 ± 0.4 [13.3; 15.0]	7.7 ± 2.7 [3.5; 14.4]

^a^Immature body and primary feather values were pooled (*n* = 3 or 4 per bird), while chick feathers are primaries only (*n* = 1 per bird).

The isotopic niche was used as proxy of the trophic niche. δ^15^N values reflect the birds' trophic position, while δ^13^C values represent their feeding habitats [8]. Whole blood and feather isotopic values reflect dietary intake in the last month and during feather synthesis, respectively. Hg is a highly toxic, non-essential metal that biomagnifies up food webs. Top predators thus accumulate Hg through food intake [9]. In the southern Indian Ocean, there is a latitudinal gradient in Hg transfer to predators, with wandering albatrosses feeding in warm subtropical waters having higher blood Hg concentrations than those feeding in colder, southern waters [10]. Blood Hg burden is thus a feeding habitat proxy. Furthermore, Hg is excreted in feathers, with concentrations reflecting accumulated burdens over the inter-moult period [11]. Stable isotope, Hg and statistical analyses are presented in the electronic supplementary material.

## 3. Results

**Figure 1.**
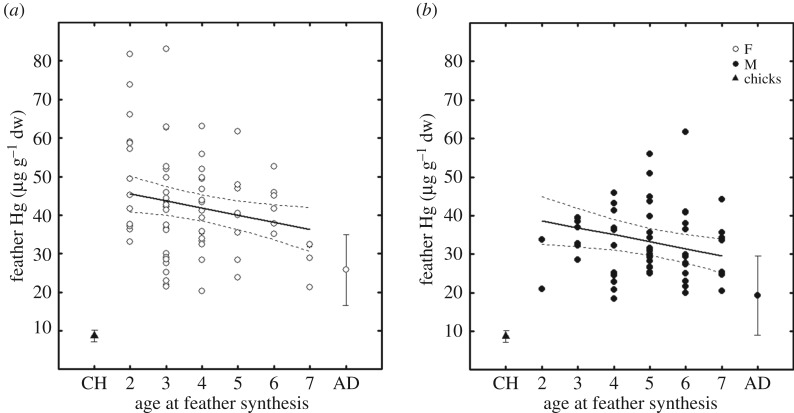
Feather Hg concentrations decrease with age in (*a*) female and (*b*) male immature wandering albatrosses from Crozet Islands. Mean ± s.d. is also presented for chicks (CH, table 1) and adults (AD) [11].

Moulting immatures fed extensively in subtropical waters, with feather δ^13^C values being generally above −18.3‰ [8]. Feather δ^13^C and δ^15^N values were correlated (table 2). Age had no effect on between-individual differences in feather δ^13^C and δ^15^N values (table 2). No gender differences were detected in feather δ^13^C and δ^15^N values (tables 1 and 2). Within-individual variation in feather δ^13^C and δ^15^N values was very high, because including a random intercept for individuals did not significantly improve model fit and explained a small part of the variance (table 2). Feather Hg concentrations decreased significantly with age in both females and males (figure 1 and table 2), with the latter having lower concentrations (tables 1 and 2). Including a random intercept for individuals improved model fit (table 2), indicating low within-individual variation in feather Hg concentrations.

**Table 2.** Effects of age and sex on feather and blood δ^13^C, δ^15^N and Hg values, and of individual random effects on feather values only, in immature wandering albatrosses from Crozet Islands. s.e., standard error; CI, 95% confidence interval; Adj., Mar. and Cond. *R*^2^, adjusted, marginal and conditional *R*^2^, respectively; LR, log-likelihood ratio.

			explained variance/deviance			effect sizes				
	model specification	model significance	Adj *R*^2^	Mar *R*^2^	Cond *R*^2^	variable	estimate ± s.e.	CI	statistics	*p*-value
feathers	linear mixed effect models, *N* = 136									
δ^15^N	∼δ^13^C + 1:Individual	LR = 85.12; *p* < 0.0001***	—	0.47	0.50	δ^13^C	0.84 ± 0.08	[0.69; 0.99]	*t* = 10.9	<0.0001***
δ^13^C	∼Age + Sex + 1:Individual	LR = 2.02: *p* = 0.364	—	0.02	0.16	Age	0.04 ± 0.05	[−0.14; 0.06]	*t* = −0.82	0.417
						Sex (M)	−0.11 ± 0.16	[−0.43; 0.21]	*t* = −0.72	0.477
						1:Individual	—	—	LR = 1.34	0.246
δ^15^N	∼Age + Sex + 1:Individual	LR = 2.25; *p* = 0.325	—	0.02	0.02	Age	−0.07 ± 0.06	[−0.18; 0.04]	*t* = −1.25	0.214
						Sex (M)	−0.03 ± 0.17	[−0.37; 0.30]	*t* = −0.20	0.844
						1:Individual	—	—	LR < 0.0001	0.999
Hg	∼Age + Sex + 1:Individual + VarPower(Age)	LR = 17.52; *p* = 0.0002***	—	0.05	0.12	Age	−1.83 ± 0.77	[−3.37; −0.30]	*t* = −2.37	0.020*
						Sex (M)	−6.82 ± 2.61	[−12.11; −1.53]	*t* = −2.61	0.013*
						1:Individual	—	—	LR = 5.70	0.017*
blood	linear (LM) or generalized linear (GLM), *N* = 39									
δ^15^N	∼δ^13^C (LM)	*F*_1, 37_ = 40.26; *p* < 0.0001***	0.51	—	—	δ^13^C	0.55 ± 0.09	[0.37; 0.72]	*t* = 6.35	<0.0001***
δ^13^C	∼Age + Sex (GLM, Gaussian identity)	Dev = −1.15; *p* = 0.178	0.09	—	—	Age	−0.05 ± 0.07	[−0.19; 0.10]	*t* = −0.61	0.544
						Sex (M)	−0.26 ± 0.22	[−0.69; 0.17]	*t* = −1.19	0.243
δ^15^N	∼Age + Sex (GLM, Gaussian identity)	Dev = −1.55; *p* = 0.010**	0.20	—	—	Age	−0.10 ± 0.05	[−0.20; 0.003]	*t* = −1.91	0.064
						Sex (M)	0.16 ± 0.15	[−0.46; 0.14]	*t* = −1.05	0.303
Hg	∼Age + Sex (GLM, Gamma inverse)	Dev = −1.24; *p* = 0.004**	0.23	—	—	Age	−0.004 ± 0.004	[−0.01; 0.004]	*t* = −0.95	0.350
						Sex (M)	0.04 ± 0.01	[0.02; 0.07]	*t* = 3.23	0.003**

**Figure 2.**
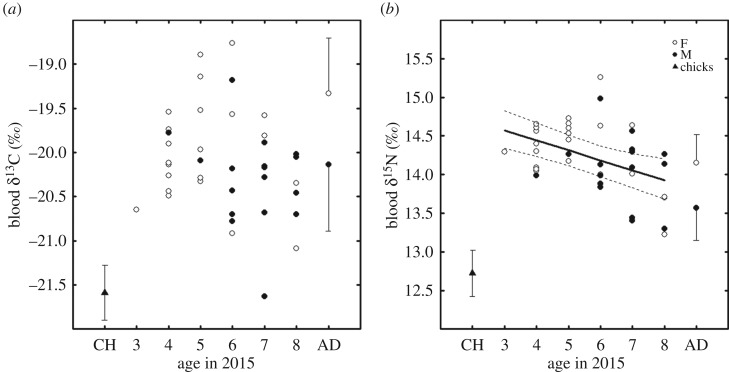
Age-related changes in blood (*a*) δ^13^C and (*b*) δ^15^N values in immature wandering albatross from Crozet Islands. Mean ± s.d. is also presented for chicks (CH, authors' unpublished data 2007) and adults (AD, young breeders) [8].

Blood δ^13^C values indicated that immature wandering albatrosses fed in subtropical (δ^13^C values > −20.1‰ [8]) and subantarctic waters (−22.9‰ < δ^13^C values < −20.1‰ [8]) while visiting Crozet Islands. Blood δ^13^C and δ^15^N values were correlated (table 2). Males had lower blood δ^13^C and δ^15^N values than females (table 1) but the difference was not significant (table 2). Interestingly, blood δ^15^N values decreased with age (figure 2), but the relationship was marginally non-significant, as shown by the small effect size (table 2). By contrast, blood δ^13^C and Hg values were not age-related (table 2). Blood Hg concentrations were significantly higher in females than in males (tables 1 and 2).

## 4. Discussion

This study presents novel trophic ecology data of the understudied yet crucial immaturity stage of a long-lived, wide-ranging seabird. Critically, results suggest that feeding habits during central-place foraging changed in a progressive manner over immaturity, and that effort should be made to study prey choice of immature seabirds as they age.

### 4.1. Moulting feeding habits

During breeding, wandering albatrosses feed extensively on squids, and rely also on fish and carrion [8,12]. Yet, very little is known on their diet outside the breeding period and during immaturity. This study confirms that moulting immatures fed extensively in subtropical waters, irrespective of gender, and occupied a very high trophic position [8]. Moreover, we showed that both between- and within-individual variations in isotopic niche were high, suggesting that immatures varied largely in their feeding habits during moult. This is consistent with the large number of oceanic destinations visited by juvenile wandering albatrosses during their first year at sea, with some individuals showing multiple strategies [13]. Between-individual variation in feeding strategies is high in all age classes [8], but adult wandering albatrosses show long-term fidelity to foraging grounds and dietary specialization [14,15]. Although tracking investigations should complement this study in order to understand subtler ontogenetic changes in feeding ecology, our results suggest that individuals continue their wide range exploration during immaturity following the juvenile stage, whereas fidelity to specific moulting grounds or dietary specialization takes place during the transition phase to maturity, or later during adulthood.

### 4.2. Feeding habits during summer

Trophic segregation of immature and breeding individuals has often been documented and could be the result of inferior foraging skills of immatures, and/or competition with adults [4,8]. While at Crozet Islands, immatures perform short foraging trips in waters surrounding the colony, where the competition with breeding adults might be high [5]. Indeed, young immatures here had higher blood δ^15^N values than breeding individuals (data from a previous study on the same population [8]), but there was a decreasing trend towards lower values as they aged (figure 2). The small size effect of this age-related decrease is likely due to the small sample size. Immatures showed also an age-related decrease, although not significant, in blood δ^13^C values. Hence, immatures progressively relied on lower trophic level prey and/or exploited colder, subantarctic waters as they aged, until having a similar niche to that of adults. These results support the progressive ontogenetic niche shift hypothesis during central-place foraging. Increasing foraging and/or competition skills of immatures as they age might enable them to select and/or access higher-quality habitats and prey around the colony. This could be critical to manage the constraints of central-place foraging and reach the body condition necessary for breeding onset [3]. The age-related niche shift was evident in blood (central-place foraging niche), but not in feathers (moulting niche), but this is not in opposition with the progressive ontogenetic niche shift hypothesis, because immatures are not exposed to the same constraints when they are central-place foragers and when they moult. Indeed, when not bound to the colony, both juveniles [13] and adults [14] disperse over huge distances, with high between-individual variation in oceanic destinations, which likely weakens intra-specific competition. The higher competition pressure around the breeding colony likely drives the ontogenetic niche shift more clearly than during moult.

### 4.3. Further insights from Hg exposure

Immature wandering albatrosses had extremely high feather and blood Hg concentrations, in agreement with their high trophic position and subtropical feeding habitats [11,16]. High Hg burdens also stem from the albatrosses' protracted moult cycle, which results in smaller Hg quantities that are excreted each year [11,16]. This mechanism could explain the age-related decrease in Hg concentrations described here. Moult is energetically demanding, thus in conflict with maintenance in young individuals, limiting the amount of feathers that can be replaced each year [7]. Over the immaturity period, with increasing foraging skills, immatures may become more efficient in energy intake and allocation, and feather replacement more regular, implying a decrease in Hg body burdens. Moreover, during visits to the natal colony, immatures gradually relied more on subantarctic waters, where Hg exposure is lower [10], thus boosting the age-related Hg decrease. Males had lower Hg concentrations than females, indicating that they spent more time in subantarctic waters. Males' preference for cold, southern latitudes could thus occur earlier than previously thought [8]. Alternatively, the larger size of males [6,7] could account for a greater dilution of Hg body burdens, or they could be physiologically less prone to Hg accumulation than females. Hg concentrations were higher than past reports, especially in females [10,11], suggesting a temporal increase in Hg bioavailability in the southern Indian Ocean, and/or a trophic shift to more Hg-contaminated prey. Young individuals are more sensitive than adults to environmental stressors, including contaminants [9]. Monitoring Hg exposure and toxic effects in immature wandering albatrosses should thus be a research priority.

## Supplementary Material

Trophic tracers quantification and statistical analyses

## Supplementary Material

Raw data

## Data accessibility

Raw data are available as electronic supplementary material.
