# Supplementary material for: Progressive ontogenetic niche shift over the prolonged immaturity period of wandering albatrosses
Source: R Soc Open Sci. 2017 Oct 11;4(10):171039. doi: 10.1098/rsos.171039 (PMC5666281; doi:10.1098/rsos.171039)
Supplement: Trophic tracers quantification and statistical analyses [file rsos171039supp1.docx]

**Supplementary material**

Progressive ontogenetic niche shift over the prolonged immaturity period of wandering albatrosses

Alice Carravieri*, Henri Weimerskirch, Paco Bustamante and Yves Cherel

Number of pages: 2; Number of sections: 2.

1. *Stable isotope and Hg determination in blood and feathers*

The relative abundance of stable isotopes was determined in feathers (homogenised by cutting them into small fragments) and lyophilised whole blood (hereafter blood) with a continuous flow isotope ratio mass spectrometer (Thermo Scientific Delta V Advantage) coupled to an elemental analyser (Thermo Scientific Flash EA 1112) (aliquots mass: ~0.3 mg) at the laboratory LIENSs, La Rochelle, France. Results are in the usual δ notation relative to Vienna PeeDee Belemnite and atmospheric N_2_ for δ^13^C and δ^15^N, respectively. Replicate measurements of internal laboratory standards (acetanilide) indicate measurement errors < 0.15 ‰ for both δ^13^C and δ^15^N values. Results are given in ‰ as means ± SD. Total mercury (Hg) was quantified with an Altec AMA 254 spectrophotometer (aliquots mass: 2-5 mg dry weight, dw). All analyses were repeated in duplicate-triplicate until having a relative standard deviation < 5% for each individual. Accuracy was checked using a certified reference material (CRM, Tort-2 Lobster Hepatopancreas, NRC, Canada; certified Hg concentration: 0.27 ± 0.06 μg g^-1^ dw). Our measured values were 0.28 ± 0.02 µg g^-1^ dw, N = 23. Blanks were analysed at the beginning of each set of samples and the limit of detection was 0.005 μg g^-1^ dw.

1. *Statistical analyses*

All statistical analyses were conducted using R 3.3.2 (R Core Team 2016). Model assumptions were checked by residuals analyses. In a first step, the effect of age and sex on δ^13^C, δ^15^N and Hg values was quantified in feathers by using linear mixed effects models (LME, package nlme, function lme), with a random intercept for Individuals. The significance of within-individual variation was tested by comparing models with and without Individual as a random effect using likelihood ratio tests (LRTs). Furthermore, we calculated the marginal and conditional R^2^, which represent the amount of variation explained by the fixed effects and by the fixed and random effects together, respectively, following Nakagawa and Schielzeth (2013). Since feather Hg variance decreased with age, the model’s variance structure was constrained to account for age (VarPower function, power = -0.57; LRT between models including or not the VarPower variance structure: likelihood ratio (LR) = 9.97, *p* = 0.002). The correlation between feather δ^13^C and δ^15^N values was also tested using a LME with a random intercept for Individual. In a second step, the effect of age and sex on δ^13^C, δ^15^N and Hg values was quantified in blood by using generalised linear models (GLM) with a gaussian (δ^13^C, δ^15^N) or a Gamma (inverse link function, Hg) family. The correlation between blood δ^13^C and δ^15^N values was tested using a linear model (LM). All models were fitted with maximum likelihood (ML) and compared against an intercept-only model using LRTs. In order to estimate effect sizes, models were then refitted using restricted maximum likelihood (REML). Age and sex (with no interaction, due to small sample sizes) were included simultaneously in all models, in order to account for the unbalanced sample size (more females were sampled at age 3 and 4, and more males at age 7 and 8).

**References**

R Core Team 2016 R: A language and environment for statistical computing. R Foundation for Statistical Computing, Vienna, Austria. URL. <https://www.R-project.org/>.

Nakagawa S, Schielzeth H 2013 A general and simple method for obtaining R^2^ from generalized linear mixed-effects models. *Methods Ecol Evol* 4:133–142
